# Thermochemical Research on Furfurylamine and 5-Methylfurfurylamine: Experimental and Computational Insights

**DOI:** 10.3390/molecules29122729

**Published:** 2024-06-07

**Authors:** Luísa M. P. F. Amaral, Ana R. R. P. Almeida, Manuel A. V. Ribeiro da Silva

**Affiliations:** 1 LAQV/REQUIMTE (Laboratório Associado para a Química Verde), Department of Chemistry and Biochemistry, Faculty of Sciences, University of Porto, Rua do Campo Alegre, P-4169-007 Porto, Portugal; 2 Research Centre in Chemistry (CIQUP), Institute of Molecular Sciences (IMS), Department of Chemistry and Biochemistry, Faculty of Sciences, University of Porto, Rua do Campo Alegre, P-4169-007 Porto, Portugal; ana.figueira@fc.up.pt (A.R.R.P.A.)

**Keywords:** furfurylamine, 5-methylfurfurylamine, energy of combustion, enthalpy of vaporization, enthalpy of formation, static bomb combustion calorimetry, Calvet microcalorimetry, G3 calculations

## Abstract

The need to transition from fossil fuels to renewables arises from factors such as depletion, price fluctuations, and environmental considerations. Lignocellulosic biomass, being abundant, and quickly renewable, and not interfering with food supplies, offers a standout alternative for chemical production. This paper explores the energetic characteristics of two derivatives of furfural—a versatile chemical obtained from biomass with great potential for commercial sustainable chemical and fuel production. The standard (*p*° = 0.1 MPa) molar enthalpies of formation of the liquids furfurylamine and 5-methylfurfurylamine were derived from the standard molar energies of combustion, determined in oxygen and at *T* = 298.15 K, by static bomb combustion calorimetry. Their standard molar enthalpies of vaporization were also determined at the same temperature using high-temperature Calvet microcalorimetry. By combining these data, the gas-phase enthalpies of formation at *T* = 298.15 K were calculated as −(43.5 ± 1.4) kJ·mol^−1^ for furfurylamine, and −(81.2 ± 1.7) kJ·mol^−1^ for 5-methylfurfurylamine. Furthermore, a theoretical analysis using G3 level calculations was performed, comparing the calculated enthalpies of formation with the experimental values to validate both results. This method has been successfully applied to similar molecules. The discussion looks into substituent effects in terms of stability and compares them with similar compounds.

## 1. Introduction

For centuries, fossil fuels have been indispensable in meeting humanity’s energy and chemical demands. However, this heavy reliance has raised concerns about the eventual depletion, supply chain disruptions, price volatility, and environmental harm from greenhouse gas emissions and pollution. Increasing fossil fuel costs have diminished their competitiveness against renewable alternatives like biomass. Thus, transitioning to renewable energy is crucial for sustainable development [1,2]. Recently, biomass has garnered attention as a plentiful renewable energy source, offering a viable alternative to traditional fossil fuels. It includes various organic materials like plants, crops, trees, agricultural residues, forestry waste, food waste, animal by-products, and human waste, which are all renewable within relatively short timeframes. Biomass is classified as a short-cycle carbon system due to its quicker replenishment compared to long-cycle carbon materials like fossil fuels. Moreover, biomass is considered carbon neutral because plants absorb CO_2_ during growth, whether from natural or human sources [3,4,5,6].

Among the biomass types, lignocellulosic biomass is particularly advantageous for chemical production. It avoids competition with food production, has a stable and abundant supply, grows rapidly at a low cost, and provides a green platform for valuable product development akin to fossil fuel-based compounds. The process of producing chemicals from biomass involves converting it into intermediate compounds called platform chemicals [7,8]. These intermediates are then refined through biological, chemical, or thermochemical methods to yield diverse and valuable compounds similar to those derived from petroleum. Furfural (FF, 2-furaldehyde) is a notable platform molecule with significant potential for commercialization in sustainable chemical and fuel production due to its versatile properties. It is a crucial foundational element that was included in the “Top10+” chemicals recognized by the US Department of Energy (DOE) in 2004. It serves as a good solvent and has found applications as a renewable resource in various industries including biofuels, detergents, lubricants, polymers, resins, food additives, pharmaceuticals (such as antiseptics and disinfectants), and agrochemicals (like herbicides, insecticides, and pesticides) [2,4,8,9]. It plays a fundamental role in the production of furan bio-based chemicals such as furfurylamine, which is essential in the creation of chemical intermediates, bioactive compounds, and medicines, including antiseptic agents, antihypertensives, and diuretics [4,9,10].

Traditionally, furfurylamine is synthesized from furfural through a chemical amination process that requires high temperatures and pressures, along with costly catalysts, often leading to environmental pollution [11]. In contrast, the biological amination of furfural has garnered significant interest due to its mild operating conditions, straightforward reaction pathway, high catalytic efficiency, low toxicity, and eco-friendly nature [12]. Apart from their pharmaceutical and agricultural uses, furfurylamine has found application in corrosion inhibitors and polymer manufacturing [13,14]. Recognized as a significant bio-based compound, furfurylamine is increasingly valued for its potential as a fuel additive due to its notable antiknock properties, positioning it as a promising enhancer for high-octane gasoline [15,16]. Through a methylation reaction involving furfurylamine, 5-methylfurfurylamine can be synthesized, which serves as a versatile building block for chemical synthesis. This compound can undergo diverse reactions, leading to functionalized derivatives with applications across pharmaceuticals and materials science [17,18,19]. Moreover, it plays a crucial role in synthesizing the new furfurylamine-derived Schiff base ligand (*E*)-4-chloro-2-((((5-methylfuran-2-yl)methyl)imino)methyl)phenol (CFMP), which has been tested as a chemosensor for recognizing metal ions [20].

**Figure 1:**
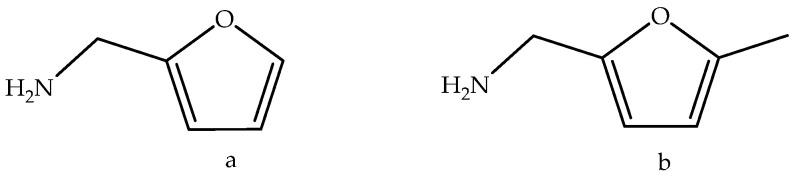
Structural formulae of (**a**) furfurylamine and (**b**) 5-methylfurfurylamine.

Despite the importance of these compounds, fundamental data on their thermodynamic properties are often scarce, hindering a detailed understanding of their energy characteristics. Continuing from previous investigations concerning furan derivatives, [21,22,23,24], this study aimed to fill this gap by measuring the standard (*p*^o^ = 0.1 MPa) molar enthalpies of combustion at 298.15 K for the liquids furfurylamine and 5-methylfurfurylamine, whose structures are represented in Figure 1, using static bomb combustion calorimetry. Additionally, their standard molar enthalpies of vaporization were determined using Calvet microcalorimetry. These experimental values were then used to calculate the gas-phase standard molar enthalpies of formation, which were compared with theoretical results obtained through high-level ab initio calculations at the G3 level of theory.

The resulting data on the energetic aspects of furfurylamine methylation offers insights into the energetic changes associated with this process. Moreover, comparisons of methylated furan and thiophene provide further context on the energetic effects of methylation, contributing to a deeper understanding of these compounds’ thermochemical behaviors.

## 2. Results

### 2.1. Experimental Results

The individual values of the massic energy of combustion for furfurylamine and 5-methylfurfurylamine, Δ_c_*u*^o^, together with their mean value, $\langle \Delta_{c}u^{o}\rangle$ , and standard deviation are presented in Table 1. Detailed values of all the combustion experiments performed for the two studied compounds are given in Tables S1 and S2 in the Supplementary Materials. These massic energy of combustion values correspond to the theoretical combustion reactions (1) and (2), which yield CO_2_ (g) and H_2_O (l).

C_5_H_7_NO (l) + 6.25 O_2_ (g) → 5 CO_2_ (g) + 3.5 H_2_O (l) + 0.5 N_2_ (g) (1)

C_6_H_9_NO (l) + 7.75 O_2_ (g) → 6 CO_2_ (g) + 4.5 H_2_O (l) + 0.5 N_2_ (g) (2)

When the samples are ignited at *T* = (298.150 ± 0.001) K

(3) $$\Delta U\left(\mathrm{IPB}\right)=-\{\varepsilon_{\mathrm{cal}}+\Delta m(H_{2}O)\cdot c_{p}\;(H_{2}O,\;l)+\varepsilon_{f}\}\;\Delta T_{\mathrm{ad}}+\Delta U\left(\mathrm{ign}\right),$$

where ΔU(IPB) is the energy associated with the isotherm bomb process, Δ*m*(H_2_O) is the deviation of mass of water added to the calorimeter from 3119.6 g, *ε*_cal_ is the energy equivalent, $\varepsilon_{f}$ is the energy of the bomb contents after ignition, Δ*U*(ign) is the ignition energy, and Δ*T*_ad_ is the adiabatic temperature rise.

The derived standard molar energies and enthalpies of combustion as well as the standard molar enthalpies of formation for the liquid compounds at *T* = 298.15 K are presented in Table 2. In accordance with normal thermochemical practice [25,26], the uncertainties assigned to the standard molar energies and enthalpies of combustion are, in each case, twice the overall standard deviation of the mean and include the uncertainties in calibration and in the values of auxiliary quantities used.

To derive $\Delta_{f}H_{m}^{o}(l)$ from $\Delta_{c}H_{m}^{o}(l)$ , the standard molar enthalpies of formation of CO_2_ (g) and H_2_O (l) at *T* = 298.15 K, (–393.51 ± 0.13) kJ·mol^−1^ [27] and (–285.830 ± 0.040) kJ·mol^−1^ [27], respectively, were used.

Table 3 provides the results of the standard molar enthalpies of vaporization ( $\Delta_{l}^{g}H_{m}^{o}$ ) determined by high-temperature Calvet microcalorimetry. The reported values for each compound at the experimental temperature *T* represent the mean values obtained from six independent experiments. The uncertainties associated with these measurements are indicated by their respective standard deviations of the mean. 

### 2.2. Computational Results

**Figure 2:**
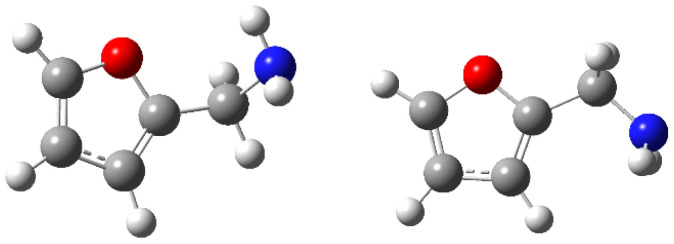
Structures of *gauche* and *syn* forms of furfurylamine.

The structure and conformation of furfurylamine were elucidated through gas-phase electron diffraction and theoretical calculations reported in the literature [28]. The analysis revealed a mixture of two conformers characterized by distinct CCCN torsion angles (φ). At 298 K, the predominant conformer (87%) adopted a *gauche* conformation with φ = 114(1)°, while the remaining molecules (13%) exhibited a *syn* conformation, where the CN bond is aligned with the carbon–carbon double bond of the furan ring (φ = 0°) (Figure 2). Both conformers could be stabilized by hydrogen bonds formed between the hydrogen atoms of the amine and either the oxygen atoms of the furan ring or the π-electrons of the carbon–carbon double bond. 

The energy and geometry of the most stable *syn* and *gauche* conformers of furfurylamine and 5-methylfurfurylamine were optimized using the G3 method. These conformations exhibit different energies due to steric hindrance. When the differences in conformational energies are sufficiently small, the likelihood of these conformations occurring in a large sample can be significant, as dictated by the Boltzmann distribution law [29]. The Boltzmann weighting factors for each conformer were determined at *T* = 298.15 K based on their relative free energies (∆G). Table S3 in the Supplementary Materials provides the relative enthalpies, free energies, and Boltzmann populations for the examined conformers. For furfurylamine, the data from Table S3 indicates a clear dominance of the *gauche* conformer, with 75.7% of the total Boltzmann relative population in the gas phase. In contrast, the *syn* conformation accounted for 24.3%. For 5-methylfurfurylamine, the Boltzmann relative populations were found to be 77.0% and 23% for the *gauche* and *syn* conformations, respectively.

Several hypothetical reactions were evaluated to estimate the gas-phase standard molar enthalpy of formation, $\Delta_{f}H_{m}^{o}\left(g\right)$ , for the compounds under investigation. The enthalpy changes of these reactions, $\Delta_{r}H_{m}^{o}\left(g\right)$ , calculated from the absolute standard enthalpies ( $H_{298.15K}^{o}$ ), were combined with the experimental $\Delta_{f}H_{m}^{o}\left(g\right)$ of all molecules involved in the reactions. This allowed us to estimate $\Delta_{f}H_{m}^{o}\left(g\right)$ at *T* = 298.15 K for the two studied compounds. The chemical reactions used to make these estimates, along with the calculated $\Delta_{r}H_{m}^{o}\left(g\right)$ values and computational estimates of $\Delta_{f}H_{m}^{o}\left(g\right)$ , are presented in Tables S4 and S5 of the Supplementary Materials. These estimates were obtained using balanced reactions that fulfil the isodesmic criterion to minimize errors caused by electronic correlation effects. Table S6 provides the G3 absolute enthalpies and experimental $\Delta_{f}H_{m}^{o}\left(g\right)$ values at *T* = 298.15 K for both compounds included in the study, along with those of the auxiliary molecules. In summary, the mean values calculated from computational estimates are collected in Table 4, along with the $\Delta_{f}H_{m}^{o}\left(l\right)$ , $\Delta_{l}^{g}H_{m}^{o}$ , and $\Delta_{f}H_{m}^{o}\left(g\right)$ values derived from experimental determinations.

## 3. Discussion

To the best of our knowledge, there are no reported thermochemistry properties for 5-methylfurfurylamine published in the literature. However, for furfurylamine, Lukyanova et al. published the result of $\Delta_{f}H_{m\;}^{o}\left(l\right)$ = −(105.4 ± 8.6) kJ·mol^−1^ [30], which differs significantly from the value derived in this study (−92.6 ± 1.1 kJ·mol^−1^). The reason for this substantial difference may lie in the amount of compound mass used by the authors in the combustion experiments. They stated that a compound mass ranging from 0.01 g to 0.02 g was used for the determinations [30], which is clearly inadequate for combustion studies and could potentially introduce errors into the measurements. 

No experimental value is known for the enthalpy of vaporization of any of the compounds studied, but it is possible to estimate this property using group contribution methods. Using the method proposed by Kolska et al. [31], the values 51.4 kJ·mol^−1^ and 53.7 kJ·mol^−1^ were obtained for furfurylamine and 5-methylfurfurylamine, respectively. According to the method presented by Naef and Acree Jr. [32], 51.6 kJ·mol^−1^ was estimated for furfurylamine and the 54.7 kJ·mol^−1^ was determined for 5-methylfurfurylamine. These results are in close agreement with the ones determined experimentally in this study. 

The crystalline structure of furfurylamine found in the literature highlights that the most significant interactions between molecules of this compound are N–H⋯N and N–H⋯O hydrogen bonds [33]. Although the crystalline pattern of 5-methylfurfurylamine was not found in the literature, the slight difference between the vaporization enthalpies of the two compounds suggest the occurrence of the same type of intermolecular interactions in the crystals of 5-methylfurfurylamine.

**Figure 3:**
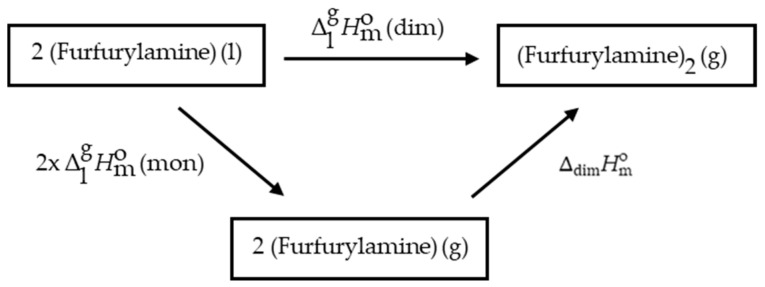
Thermochemical cycle of the vaporization of dimer and monomer of furfurylamine.

Lukyanova et al. [30] suggest that in the gas phase, furfurylamine may exist entirely in its dimeric form. If this is the case, the measured enthalpy of vaporization would not correspond to that of the monomeric form. To determine whether dimerization occurs in the gas phase, we can analyze the thermochemical cycle illustrated in Figure 3.

From this thermochemical cycle, the standard molar enthalpies of vaporization can be derived using Equations (4) and (5) for the monomer and the dimer, respectively. Table 5 presents the calculated values for the enthalpies of vaporization. These values assume $\Delta_{f}H_{m}^{o}\left(g\right)$ = −38.9 kJ·mol^−1^ and use the experimental values for $\Delta_{f}H_{m\;}^{o}(l)\;$ determined in this study and in reference [30] and ${∆}_{\dim}H_{m\;}^{o}\;$ = −24 kJ·mol^−1^ [30]. As we can demonstrate through our calculations, the experimental value determined in this work, $\Delta_{l}^{g}H_{m}^{o}$ (exp) = 49.1 kJ·mol^−1^, is close to that of the monomeric form.

(4) $$\Delta_{l}^{g}H_{m}^{o}\left(\mathrm{mon}\right)=\Delta_{f}H_{m\;}^{o}\left(g\right)-\Delta_{f}H_{m\;}^{o}\left(l\right)\;$$

(5) $${∆}_{l}^{g}H_{m}^{o}\left(\dim\right)={2∆}_{f}H_{m\;}^{o}(g)\;-{2∆}_{f}H_{m\;}^{o}\left(l\right)+{∆}_{\dim}H_{m\;}^{o}$$

The mean theoretical estimates (Table 4) show good agreement with the experimental data, with a maximum deviation of only 4.6 kJ·mol^−1^ within the range of uncertainties of 4–5 kJ·mol^−1^, a tolerance commonly referred to as “chemical accuracy”. This consistency highlights that the reliability of the composite G3 method with the isodesmic reaction yields precise thermochemical data for such compounds. Thus, we can confidently accept the theoretical estimates. 

The knowledge of $\Delta_{f}H_{m}^{o}\left(g\right)$ values for chemical compounds is crucial in various fields of chemistry, providing insights into their thermodynamic stability, reactivity, and energetic properties. In particular, the knowledge of these values for O or S five-membered rings holds significant importance due to the widespread applications of furan/thiophene-based compounds in pharmaceuticals, materials science, and organic synthesis.

**Figure 4:**
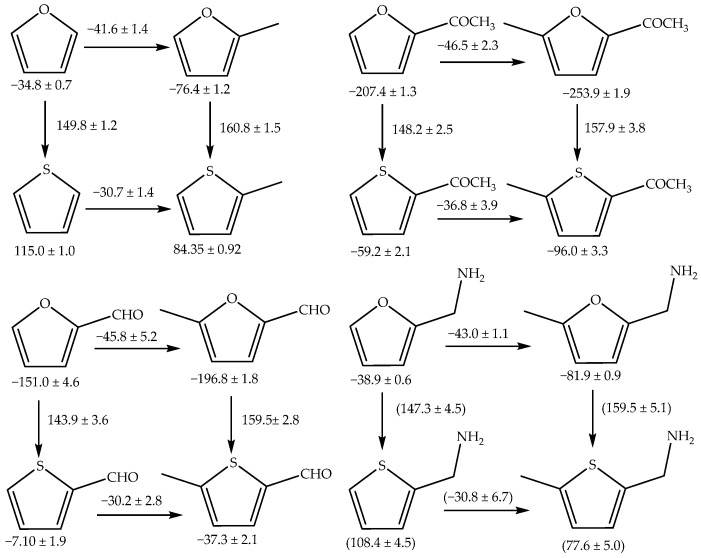
Enthalpic increments (values in kJ·mol^−1^) for the introduction of a methyl group in furan and thiophene derivatives. Enthalpy of oxygen to sulfur substitution.

Using values of $\Delta_{f}H_{m}^{o}\left(g\right)$ collected from literature [21,23,34,35,36], it is possible to evaluate the enthalpic contribution of the methyl group as a substituent on the structure of furan and to compare it with the effects observed for identical substitutions on thiophene derivatives, as can be seen in the scheme in Figure 4. 

Comparing the introduction of a methyl group into furan versus thiophene, it becomes evident that the process is slightly more favorable in furan. This observation likely stems from the structural and electronic differences between the two heterocycles where the electronegativity difference between oxygen and sulfur plays a crucial role. Oxygen is more electronegative than sulfur, leading to a greater partial positive charge on the carbon atoms adjacent to the heteroatom in furan compared to thiophene. This increased positive charge in furan makes it more susceptible to nucleophilic attack, thereby favoring the addition of a methyl group. Moreover, the enthalpy associated with substituting an oxygen atom with a sulfur atom has been quantified in substituted furans, including derivatives like furan-2-aldehyde (−CHO) and 2-acethylfuran (−COCH₃), as well as methylfurans. The enthalpy change for this substitution process is approximately (147.3 ± 4.5) kJ·mol^−1^ for substituted furans and slightly higher, at (159.4 ± 5.0) kJ·mol^−1^, for 5-methylfurans. Assuming that these enthalpic incremental changes due to oxygen → sulfur substitution will be the same for furfuryl derivatives, we can estimated that $\Delta_{f}H_{m}^{o}\left(g\right)$ = (108.4 ± 4.5) kJ·mol^−1^ and $\Delta_{f}H_{m}^{o}\left(g\right)$ = (77.6 ± 5.0) kJ·mol^−1^ for the compounds 2-thiophenemethylamine and 5-methyl-2-thiophenemethylamine, respectively.

The calculated enthalpic increments for the methylation reactions in this study ranged from −38.4 to −46.5 kJ·mol^−1^ for furan and from −30.2 to −36.8 kJ·mol^−1^ for thiophene. According to Freitas et al. [37,38], the enthalpic increments for the insertion of a single methyl group in various positions of benzofuran are between −30.3 and −36.2 kJ·mol^−1^ [37], while for benzothiophene, the increments range from −31.1 to −38.0 kJ·mol^−1^ [38]. For dibenzofuran, the same authors reported an interval of −30.5 to −37.0 kJ·mol^−1^ [37] for the addition of a methyl group in different positions, and for dibenzothiophene, the range is −29.5 to −39.1 kJ·mol^−1^ [38]. These values are similar to those found for the inclusion of methyl groups into benzene or naphthalene, which fall in the interval of −31.0 to −35.0 kJ·mol^−1^ [34]. Except for furan, the effect of methylation at different positions of the heterocyclic compounds benzofuran, dibenzofuran, thiophene, and benzothiophene is to decrease the enthalpy of formation by −30 to −39 kJ·mol^−1^. In the case of furan, this stabilization is higher, which agrees with the results of Simmie et al. [39], who reported similar deviations for various other species such as 2-hydroxyfuran (−42.9 kJ·mol^−1^), 2-furaldehyde (−48.3 kJ·mol^−1^), 2-furancarboxilic acid (−47.4 kJ·mol^−1^), and 2-methoxyfuran (−43.4 kJ·mol^−1^). Even methylation of 5-methyl-3-furoic acid to 2,5-dimethyl-3-furoic acid follows a similar course [39]. The authors also added that recent theoretical works on the enthalpies of formation of furan, 2-methylfuran, and 2,5-dimethylfuran allowed them to estimate an increment of −45.5 and −44.3 kJ·mol^−1^ for this system while the increments relative to the similar scheme for thiophene, 2-methylthiophene, and 2,5-dimethylthiophene amounted to −31.5 and −32.9 kJ·mol^−1^, respectively [39].

Another useful comparison involves the transfer of a –CH_2_NH_2_ substituent from furan to thiophene in the isodesmic reaction (6), which allows for the prediction of $\Delta_{f}H_{m}^{o}\left(g\right)$ for 2-thiophenemethylamine to be 120.2 kJ·mol^−1^. Likewise, using the isodesmic reaction (7), we can estimate the value of $\Delta_{f}H_{m}^{o}\left(g\right)\;$ to be 87.3 kJ·mol^−1^ for 5-methyl-2-thiophenemethylamine.
(6)
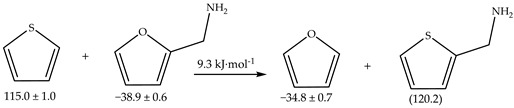

(7)
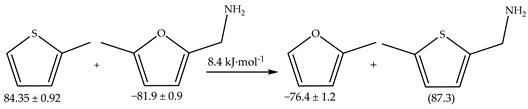


The use of computational and/or group contributions methods to determine thermodynamic properties must be carried out with caution. This is because both are based on experimental results that are not always well determined due to deficiencies in the techniques or procedures used or even due to the inadequate treatment of the results. To avoid this situation, the experimental values reported in the literature for the molecules used in these methods must be carefully selected. This selection is based on the knowledge and confidence in the results of some research groups with extensive experience in the experimental determination of thermochemical properties and on the recurrent and successful use of the selected values in numerous reactions aimed at estimating the thermodynamic properties of very different organic compounds used in previous works. When suitable experimental reference values are available, we may get a good estimate for the absolute value.

## 4. Materials and Methods

### 4.1. Materials and Purity Control

The furfurylamine (CAS 617-89-0) and 5-methylfurfurylamine (CAS 14003-16-8) studied in this work were acquired commercially from Aldrich Chemical Co. (St. Louis, MO, USA) with mass fraction purities of 0.99 and 0.97, respectively. Both compounds were subject to purification through vacuum distillation, under reduced pressure, until consistent combustion results were achieved. The purity of the compounds was verified using gas–liquid chromatography on an Agilent 4890D Gas Chromatography system, (Santa Clara, CA, USA) featuring an HP-5 column with a composition of 5% diphenyl and 95% dimethylpolysiloxane (15 × 0.530 mm i.d × 1.5 μm film thickness). Nitrogen served as the carrier gas, and the purity was confirmed to be ≥0.9995 mass fraction. Additional details regarding the origin and purification of the samples are provided in Table S7 in the Supplementary Materials. 

The final purity of each sample was asserted during the combustion experiments by the proximity of the carbon dioxide recovery ratios to unity. 

### 4.2. Combustion Calorimetry

The combustion experiments were performed with a static bomb combustion calorimeter, using a twin valve bomb, type 1108, from the Parr Instrument Company (Moline, IL, USA). The apparatus and technique have been described previously [40,41] so only a brief description is given here.

For the calibration of the bomb, we used certificated benzoic acid, NBS Standard Reference Material, Sample 39j, whose massic energy of combustion is −(26,434 ± 3) J·g^−1^ under certificate conditions [42]. The calibration results were corrected to give the energy equivalent *ε*_cal_ corresponding to the average mass of water added to the calorimeter: 3119.6 g. For the study of furfurylamine, *ε*_cal_ = (15,907.1 ± 0.7) J·K^−1^, and for the study of 5-methylfurfurylamine, *ε*_cal_ = (15,917.0 ± 1.4) J·K^−1^, where the uncertainty quoted is the standard deviation of the mean. The combustion experiments were performed in oxygen at *p* = 3.04 MPa, with 1.00 cm^3^ of water added to the bomb. The bomb was purged twice to remove air before being charged with oxygen. The electrical energy for ignition, Δ*U*(ign), was determined from the change in potential difference across a capacitor when discharged through the platinum ignition wire. 

For all experiments, the calorimeter temperatures were measured to ±1 × 10^–4^ K, at time intervals of 10 s, with a quartz crystal thermometer (Hewlett Packard HP 2804A), (Palo Alto, CA, USA), interfaced to a PC. At least 100 readings of the temperature were taken before the ignition of the samples which occurred at *T* = (298.150 ± 0.001) K using the discharge of a 1400 μF capacitor through a platinum ignition wire. After ignition, 100 readings were taken for the main and the periods after.

The liquid samples were contained in sealed polyester bags made of Melinex^®^ (New Berlin, WI, USA), (0.025 mm of thickness) with a massic energy of combustion Δ_c_*u*^o^ = –(22,902 ± 5) J·g^–1^ [43], a value which was confirmed in our laboratory. The mass of Melinex^®^ used in each experiment was corrected for the mass fraction of water (*w* = 0.0032). For the cotton-thread fuse, with empirical formula CH_1.686_O_0.843_, the value of Δ_c_*u*^o^ = –16,240 J·g^−1^ [44] was taken for the massic energy of combustion, a value that was confirmed in our laboratory. 

The corrections for nitric acid formation were based on −59.7 kJ·mol^−1^ [45] for the molar energy of formation of 0.1 mol·dm^−3^ HNO_3_(aq) from N_2_, O_2_, and H_2_O(l). All the necessary weighing was performed in a Mettler Toledo AT201 microbalance, with a sensitivity of ±(1 · 10^–6^) g, and corrections of the apparent mass to the true mass were made. The estimated pressure coefficient of specific energy, *(∂u/∂p)T* = –0.2 J·g^−1^·MPa^−1^ at *T* = 298.15 K, is a typical value for most organic compounds [46]. For each compound, the massic energy of combustion, Δ_c_*u*^o^, was calculated using the procedure given by Hubbard et al. [47]. The quantity of compound used in each experiment was calculated based on the total mass of carbon dioxide produced during the combustion experiments. This calculation accounted for the carbon dioxide generated from the combustion of the cotton-thread fuse and from the Melinex^®^. Carbon dioxide was collected in absorption tubes containing Ascarite (sodium hydroxide-coated silica) that were pre-weighed.

The densities at *T* = 298.15 K were measured for furfurylamine (1.099 g∙cm^−3^) [48], for 5-methylfurfurylamine (0.997 g∙cm^−3^) [48], for the cotton-thread fuse (*ρ* = 1.50 g·cm^−3^) [47] and for Melinex^®^ (*ρ* = 1.38 g·cm^−3^) [43]. Specific heat capacities of *c_p_* = 1.3 J·g^−1^·K^−1^ and *c_p_* = 1.67 J·g^−1^·K^−1^ were used for Melinex^®^ and cotton, respectively [47]. The value for the pressure coefficient of massic energy, (*∂u*/*∂p*)*_T_*, for Melinex^®^ was –0.03 J·g^−1^·MPa^−1^; for cotton, it was –0.29 J·g^−1^·MPa^−1^ [47]; and for the studied compounds, it was assumed to be –0.2 J·g^−1^·MPa^−1^ at *T* = 298.15 K, which is a typical value for most organic compounds [46]. The relative atomic masses used were those recommended by the IUPAC Commission in 2013 [49].

### 4.3. High Temperature Microcalorimetry

A Calvet High Temperature Microcalorimeter (Setaram, HT 1000D) [50] was employed to determine the standard molar enthalpies of vaporization for the liquid compounds. This method is similar to the technique described for the sublimation of solids [51]. Samples of about 3 to 7 mg of liquid compound, contained in a small thin glass capillary tube sealed at one end, and a blank capillary, were simultaneously dropped at room temperature into the hot reaction vessels in the Calvet high-temperature microcalorimeter held at a predefined temperature, *T*, and were removed from the hot zone by vacuum evaporation. The thermal correction for the glass capillary tubes was established through separate experiments. In each experiment, the thermal corrections were minimized by carefully selecting tubes with nearly identical masses, within a range of ±10 µg.

The observed enthalpy change, $\Delta_{l,\;298.15K}^{g,T}H_{m}^{o}$ , was corrected to 298.15 K by utilizing the following equation:

(8) $$\Delta_{l}^{g}H_{m}^{o}(298.15K)=\Delta_{l,\;298.15K}^{g,T}H_{m}^{o}-\int_{298.15\;K}^{T}C_{p,m}^{o}(g)dT,$$

where *T* is the temperature of the hot calorimeter cells, and $C_{p,m}^{o}\;(g)$ = f(*T*), the temperature dependence of the molar heat capacity. We calculated the gaseous heat capacities of furfurylamine and 5-methylfurfurylamine at the B3LYP/6-31G(2df,p) level (scaled by a factor of 0.965 [52]) (see Table S8 and Equations (S2) and (S3) provided in the Supplementary Materials).

The microcalorimeter was calibrated in situ for these measurements using the reported standard molar enthalpy of vaporization of *n*-decane in the case of furfurylamine and *n*-undecane for 5-methylfurfurylamine [53].

### 4.4. Computational Approach 

We conducted accurate calculations based on the composite G3 approach [54]. Previously, the G3 method was successfully employed to estimate the enthalpies of formation of similar compounds [39,55,56]. This procedure uses geometries from second-order perturbation theory [MP2(FU)/6-31G(d)] to perform a series of very accurate single-point energy calculations at the second-order Moller–Plesset (MP2), fourth-order Moller–Plesset (MP4), and quadratic configuration interaction [QCISD(T)] levels of theory. The overall result from such calculations uses scaled zero-point energies obtained from the Hartree–Fock theory with the 6-31G(d) basis set and, by adopting certain assumptions about the additivity of the calculations and including a spin-orbit correction and a higher-level correction, aimed to effectively reproduce the results of a calculation at the QCISD(T,FU)/G3 large level. All calculations were performed using the Gaussian 09 package of programs [57].

## 5. Conclusions

The relevant thermochemical and thermodynamic properties of the bio-based platform chemicals, furfurylamine and 5-methylfurfurylamine, were determined in this work:

- Using combustion calorimetry, the enthalpies of formation in the liquid phase were derived for both compounds as −(92.6 ± 1.1) kJ·mol^−1^ for furfurylamine and −(134.5 ± 1.5) kJ·mol^−1^ for 5-methylfurfurylamine.
- The enthalpy of formation of furfurylamine in the liquid phase derived in this work is quite different from the value reported before in the literature
- Through high-temperature Calvet microcalorimetry, the enthalpies of vaporization of the compounds studied at 298.15 K were obtained: (49.1 ± 0.8) kJ·mol^−1^ for furfurylamine and (53.3 ± 0.9) kJ·mol^−1^ for 5-methylfurfurylamine.
- Combining these data, their gas-phase enthalpy of formation was determined as −(43.5 ± 14) kJ·mol^−1^ for furfurylamine, and −(81.2 ± 1.7) kJ·mol^−1^ for 5-methylfurfurylamine.
- The enthalpies of formation in the gaseous phase were also estimated by a theoretical analysis using G3 level calculations.
- An excellent agreement between the experimental and computational results of the standard enthalpy of formation in gaseous phase was achieved.
- The evaluation and the comparison of the enthalpic contribution of the methyl group as a substituent on the structure of similar compounds were performed in the gas phase.
- The enthalpies of formation in the gaseous phase of 2-thiophenemethylamine and 5-methyl-2-thiophenemethylamine were estimated using both empirical and computational methods.

## Figures and Tables

**Table 1:** Individual values of the massic energy of combustion, Δ_c_*u*^o^, of the studied compounds at *T* = 298.15 K. All values are in J·g^−1^.

Furfurylamine	5-Methylfurfurylamine
−29,591.73	−31,577.92
−29,582.70	−31,568.06
−29,589.96	−31,562.26
−29,589.56	−31,577.61
−29,593.49	−31,586.75
−29,581.42	−31,579.48
	−31,589.80
	−31,584.93
$\langle \Delta_{c}u^{o}\rangle$ ^a^	
−29,588.1 ± 2.0	−31,577.4 ± 3.3

^a^ Mean value and standard deviation of the mean.

**Table 2:** Standard (*p*^o^ = 0.1 MPa) molar energies of combustion ( $\Delta_{c}U_{m}^{o}$ ), enthalpies of combustion $\left(\Delta_{c}H_{m}^{o}\right)$ , and enthalpies of formation ( $\Delta_{f}H_{m}^{o}$ ) for the two compounds studied, at *T* = 298.15 K. ^a^ Values in kJ·mol^−1^.

Compound	$\text{–}\Delta_{c}U_{m\;}^{o}\left(l\right)$	$-\Delta_{c}H_{m\;}^{o}\left(l\right)$	$-\Delta_{f}H_{m\;}^{o}\left(l\right)$
Furfurylamine	2873.5 ± 0.9	2875.4 ± 0.9	92.6 ± 1.1
5-Methylfurfurylamine	3509.7 ± 1.3	3512.8 ± 1.3	134.5 ± 1.5

^a^ The uncertainties are twice the overall standard deviation of the mean and include the contributions from the calibration with benzoic acid and from the energy of combustion of auxiliary materials.

**Table 3:** Microcalorimetric standard (*p*^o^ = 0.1 MPa) molar enthalpies of vaporization (in kJ·mol^−1^) at *T* = 298.15 K.

Compound	${\frac{T}{K}}^{\;a}$	$\Delta_{l,\;298K}^{g,T}{H_{m}^{o}}^{\;b}$	$\Delta_{298.15K}^{T}H_{m}^{o}$	$\Delta_{l}^{g}H_{m}^{o}(298.15\;K{)}^{\;c}$
Furfurylamine	344.5	54.5 ± 0.4	5.4	49.1 ± 0.8
5-Methylfurfurylamine	339.9	59.3 ± 0.4	6.0	53.3 ± 0.9

^a^ *u*(*T*) = ±0.1 K. ^b^ Mean value and standard deviation of the mean of six experiments. ^c^ The uncertainties are twice the overall standard deviation of the mean and include the contributions from the calibration.

**Table 4:** Standard (*p*^o^ = 0.1 MPa) molar enthalpies of formation, in both liquid and gaseous phases, and standard molar enthalpy of vaporization at *T* = 298.15 K.

Compound	$\frac{\Delta_{f}H_{m}^{o}\left(l\right)}{\mathrm{kJ}\cdot \mathrm{mo}l^{-1}}$	$\frac{\Delta_{l}^{g}H_{m}^{o}}{\mathrm{kJ}\cdot \mathrm{mo}l^{-1}}$	$\frac{\Delta_{f}H_{m}^{o}\left(g\right)}{\mathrm{kJ}\cdot \mathrm{mo}l^{-1}}$	
			Experimental ^a^	G3 ^b^
Furfurylamine	–92.6 ± 1.1	49.1 ± 0.8	–43.5 ± 1.4	–38.9 ± 0.6
5-Methylfurfurylamine	–134.5 ± 1.5	53.3 ± 0.9	–81.2 ± 1.7	–81.9 ± 0.9

^a^ Uncertainties calculated through the RSS (Root-Sum-Square) method; ^b^ mean and standard deviation of the mean.

**Table 5:** Vaporization enthalpies according to Equations (4) and (5) for furfurylamine.

	$\Delta_{l}^{g}H_{m}^{o}$ /kJ·mol^−1^	
	This Work	Lukyanova et al. [30]
Furfurylamine (monomer)	53.7	66.5
Furfurylamine (dimer)	83.4	109.0

## Data Availability

The data presented in this study are available in the Supplementary Materials.

## Supplementary Materials

The following supporting information can be downloaded at: https://www.mdpi.com/article/10.3390/molecules29122729/s1, Table S1: Results of combustion experiments at *T* = 298.15 K of furfurylamine; Table S2: Results of combustion experiments at *T* = 298.15 K of 5-methylfurfurylamine; Table S3: Absolute enthalpies, $H_{298.15K}$ , Gibbs energies, $G_{298.15K}$ , relative free energies and Boltzmann populations in gas-phase, calculated at G3 level of theory. 1 a.u. (Hartree) corresponds to 2625.50 kJ·mol^−1^. *Calculated from $e^{-\left[\Delta G_{\mathrm{rel}}/RT\right]}/\sum_{i}^{n}e^{-\left[\Delta G_{\mathrm{rel}}/RT\right]}$ ; Table S4: Computed enthalpies of reaction, $\Delta_{r}H_{m}^{o}$ , and formation, $\Delta_{f}H_{m}^{o}$ , in the gaseous state, of furfurylamine, using G3 at *T* = 298.15 K; Table S5: Computed enthalpies of reaction, $\Delta_{r}H_{m}^{o}$ , and formation, $\Delta_{f}H_{m}^{o}$ , in the gaseous state, of 5-methylfurfurylamine, using G3 at *T* = 298.15 K; Table S6: G3 computed enthalpies for furfurylamine and 5-methylfurfurylamine and for the auxiliary molecules used in the gas-phase reactions and standard molar enthalpies of formation, at *T* = 298.15 K, taken from the literature; Table S7: Purification details of furfurylamine and 5-methylfurfurylamine; Table S8: Standard molar heat capacities, in the gaseous phase, of furfurylamine and 5-methylfurfurylamine derived from statistical thermodynamics using the vibrational frequencies calculated at the basis B3LYP/6-31G(2df,p) level of theory (scaled by a factor of 0.965). References [23,34,52,58,59,60,61,62] are cited in the Supplementary Materials.
