# Correction: Amaral et al. Thermochemical Research on Furfurylamine and 5-Methylfurfurylamine: Experimental and Computational Insights. *Molecules* 2024, *29*, 2729

**DOI:** 10.3390/molecules30132773

**Published:** 2025-06-27

**Authors:** Luísa M. P. F. Amaral, Ana R. R. P. Almeida, Manuel A. V. Ribeiro da Silva

**Affiliations:** 1 LAQV/REQUIMTE (Laboratório Associado para a Química Verde), Department of Chemistry and Biochemistry, Faculty of Sciences, University of Porto, Rua do Campo Alegre, P-4169-007 Porto, Portugal; 2 Research Centre in Chemistry (CIQUP), Institute of Molecular Sciences (IMS), Department of Chemistry and Biochemistry, Faculty of Sciences, University of Porto, Rua do Campo Alegre, P-4169-007 Porto, Portugal; ana.figueira@fc.up.pt

## Error in Figure

In the original publication [1], there was an error in the Discussion section in Figure 3, entitled “*Thermochemical cycle of the vaporization of dimer and monomer of furfurylamine*”. The arrow representing ${∆}_{\mathrm{diss}}H_{m\;}^{o}$ was incorrectly labeled. The corrected Figure 3 is provided below:

## Text Correction

Additionally, there were errors in the Discussion section following Figure 3 in the original publication. In accordance with the revised Figure 3, the value ${∆}_{\mathrm{diss}}H_{m\;}^{o}\;$= 24 kJ·mol^−1^ [30] was replaced with ${∆}_{\dim}H_{m\;}^{o}\;$= −24 kJ·mol^−1^ [30].

Equation (5) of the original publication is related to Figure 3 and was therefore incorrect. The corrected Equation (5) is shown below:

(5) $${∆}_{l}^{g}H_{m}^{o}\left(\dim\right)={2∆}_{f}H_{m\;}^{o}(g)\;-{2∆}_{f}H_{m\;}^{o}\left(l\right)+{∆}_{\dim}H_{m\;}^{o}$$

## Error in Table

In the original publication, there was also an error in Table 5 (Discussion section), titled “*Vaporization enthalpies according to Equations (4) and (5) for furfurylamine*”. The values of ${∆}_{l}^{g}H_{m}^{o}$ calculated in this work for furfurylamine (dimer) were incorrect. The corrected values are now presented in the revised Table 5.

The authors state that the scientific conclusions are unaffected. This correction was approved by the Academic Editor. The original publication has also been updated.

## Figures and Tables

**Figure 3:**
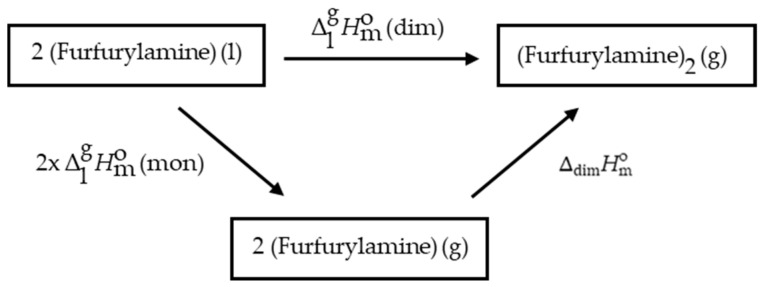
Thermochemical cycle of the vaporization of dimer and monomer of furfurylamine.

**Table 5:** Vaporization enthalpies according to Equations (4) and (5) for furfurylamine.

	$\Delta_{l}^{g}H_{m}^{o}$/kJ·mol^−1^	
	This Work	Lukyanova et al. [30]
Furfurylamine (monomer)	53.7	66.5
Furfurylamine (dimer)	83.4	109.0
