# Supplementary material for: Thermochemical Research on Furfurylamine and 5-Methylfurfurylamine: Experimental and Computational Insights
Source: Molecules. 2024 Jun 7;29(12):2729. doi: 10.3390/molecules29122729 (PMC11205687; doi:10.3390/molecules29122729)
Supplement: Supplementary file 1 [file molecules-29-02729-s001.zip › molecules-3025392-supplementary.pdf]

Supplementary Materials

Table S1. Results of combustion experiments at T = 298.15 K of furfurylamine.

**Experiment nº**	**1**	**2**	**3**	**4**	**5**	**6**
m′(cpd) / g	0.63493	0.66633	0.45132	0.69286	0.63371	0.72167
m′′(fuse) / g	0.00226	0.00250	0.00231	0.00235	0.00230	0.00269
m''(Melinex®) /g	0.05952	0.05879	0.05788	0.05778	0.06247	0.05581
ΔT_{ad} / K	1.27134	1.32875	0.92690	1.37676	1.27360	1.42755
ε_f / J·K^{-1}	16.18	16.19	15.71	16.32	16.16	16.34
$\Delta m(H_2O)$ / g	0.0	0.0	0.0	0.0	0.0	0.0
$-\Delta U$ (IBP) / J[a]	20242.94	21157.22	14757.91	21921.83	20278.87	22730.60
ΔU(Melinex®) / J	1363.15	1346.47	1325.68	1323.17	1430.77	1278.16
ΔU (fuse) / J	36.70	40.60	37.51	38.16	37.35	43.69
ΔU (HNO_3) / J	42.09	45.43	31.34	45.67	44.72	46.80
ΔU(ign) / J	0.96	0.85	0.94	0.90	0.99	0.91
ΔU_Σ / J	12.32	12.88	8.84	13.41	12.34	13.93
$-\Delta_c u^o$ / J·g^{-1}	29591.73	29582.70	29589.96	29589.56	29593.49	29581.42

$-\langle \Delta_c u^o \rangle$ = 29588.1 ± 2.0 J·g^{-1}[b]

[a] ΔU(IBP) already includes the ΔU(ign).

[b] Mean value and standard deviation of the mean.

Table S2. Results of combustion experiments at T = 298.15 K of 5-methylfurfurylamine.

**Experiment nº**	**1**	**2**	**3**	**4**	**5**	**6**	**7**	**8**
m(cpd) / g	0.60107	0.44562	0.56344	0.48083	0.50597	0.51508	0.41075	0.48409
m′(fuse) / g	0.00273	0.00286	0.00296	0.00266	0.00257	0.00283	0.00293	0.00270
m′′(Melinex®)/g	0.05266	0.05166	0.05299	0.05411	0.05599	0.05413	0.05633	0.05235
ΔT_{ad} / K	1.27283	0.96234	1.19820	1.03607	1.08872	1.10431	0.90054	1.04019
ε_f / (J·K^{-1})	16.26	15.84	16.16	15.91	16.02	16.02	15.74	15.93
$\Delta m(H_2O)$ / g	0.0	0.0	0.0	0.0	0.0	0.1	0.0	0.0
$-\Delta U$(IBP) / J[a]	20280.09	15332.46	19090.94	16507.20	17346.31	17595.02	14347.72	16572.97
ΔU(Melinex®) /J	1205.97	1183.13	1213.51	1239.33	1282.28	1239.78	1290.04	1198.89
ΔU(fuse) / J	44.34	46.45	48.07	43.20	41.74	45.96	47.58	43.85
$\Delta U(HNO_3)$ / J	38.03	27.16	35.40	32.18	30.81	33.67	26.75	31.22
ΔU(ign) / J	0.75	0.73	0.65	0.82	0.72	0.88	0.71	0.72
ΔU_{Σ} / J	11.21	8.36	10.52	9.03	9.53	9.65	7.84	9.06
$-\Delta_c u^o$ / J·g^{-1}	31577.92	31568.06	31562.26	31577.61	31586.75	31579.48	31589.80	31584.93

$-\langle \Delta_c u^o \rangle$ = 31578.4 ± 3.3 J·g^{-1}[b]

[a]ΔU (IBP) already includes the ΔU (ign).

[b]Mean value and standard deviation of the mean.

m(cpd) is the mass of compound burnt in each experiment; m'(fuse) is the mass of the fuse (cotton) used in each experiment; m′′(Melinex®) is the mass of the Melinex® bags; ΔT_{ad} is the corrected temperature rise; ε_f is the energy equivalent of the contents in the final state; $\Delta m(H_2O)$ is the deviation of mass of water added to the calorimeter from 3119.6 g; ΔU(IBP) is the energy change for the isothermal combustion reaction under actual bomb conditions; ΔU(Melinex®) is the energy of combustion of the Melinex® bags; ΔU(fuse) is the energy of combustion of the fuse (cotton); $\Delta U(HNO_3)$ is the energy correction for the nitric acid formation; ΔU(ign) is the electric energy for the ignition; ΔU_{Σ} is the standard state correction; $\Delta_c u^o$ is the standard massic energy of combustion.

Table S3. Absolute enthalpies, $H_{298.15K}$, Gibbs energies, $G_{298.15K}$, relative free energies and Boltzmann populations in gas-phase, calculated at G3 level of theory. 1 a. u. (Hartree) corresponds to 2625.50 kJ·mol⁻¹. *Calculated from $e^{-[\Delta G_{\mathrm{rel}}/RT]}/\sum_i^n e^{-[\Delta G_{\mathrm{rel}}/RT]}$

Structure	$\frac{H_{298.15K}}{\text{Hartree}}$	$\frac{G_{298.15K}}{\text{Hartree}}$	$\frac{\Delta H_{298.15K}}{\text{kJ·mol}^{-1}}$	$\frac{\Delta G_{298.15K}}{\text{kJ·mol}^{-1}}$	Boltzmann relative population (%)*
Furfurylamine					
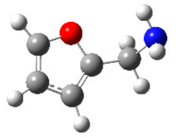 Gauche form	–324.442355	–324.480514	0.00	0.00	75.7
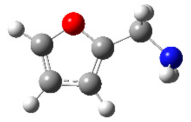 Syn form	–324.441387	–324.479443	2.54	2.81	24.3
5-Methylfurfurylamine					
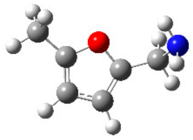 Gauche form	–363.720879	–363.762991	0.00	0.00	77.0
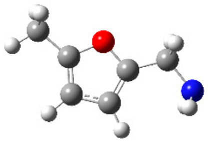 Syn form	–363.71982	–363.761852	2.78	2.99	23.0

Table S4. Computed enthalpies of reaction, $\Delta_r H^o_m$, and formation, $\Delta_f H^o_m$, in the gaseous state, of furfurylamine, using G3 at T = 298.15 K

Reaction		$\frac{\Delta_r H^o_m(g)}{kJ \cdot mol^{-1}}$	$\frac{-\Delta_f H^o_m(g)}{kJ \cdot mol^{-1}}$
Furfurylamine + 2-methylfuran → 5-methylfurfurylamine + furan	(1)	0.2	39.8
Furfurylamine + ethane → ethylamine + 2-methylfuran	(2)	–2.5	37.5
Furfurylamine + methane → ethylamine + furan	(3)	32.8	40.7
Furfurylamine + benzene → aniline+ 2-methylfuran	(4)	–35.5	36.7
Furfurylamine + benzaldehyde → benzamide + 2-methylfuran	(5)	–100.0	40.6
Furfurylamine + formaldehyde → methylamine + furaldehyde	(6)	–27.5	38.3
Furfurylamine + toluene → 2,6-dimethylaniline + furan	(7)	–27.7	38.6
		$\langle \Delta_f H^o_m(g) \rangle$ (G3) = (–38.9 ± 0.6) kJ·mol^{-1}[a]	

[a]Mean and standard deviation.

Table S5. Computed enthalpies of reaction, $\Delta_r H^o_m$, and formation, $\Delta_f H^o_m$, in the gaseous state, of 5-methylfurfurylamine, using G3 at T = 298.15 K

Reaction		$\frac{\Delta_r H^o_m(g)}{kJ \cdot mol^{-1}}$	$\frac{-\Delta_f H^o_m(g)}{kJ \cdot mol^{-1}}$
5-Methylfurfurylamine + furan → furfurylamine + 2-methylfuran	(1)	–0.2	84.9
5-Methylfurfurylamine + methane → ethylamine + 2-methylfuran	(2)	32.7	82.2
5-Methylfurfurylamine + benzene → aniline + 2,5-dimethylfuran	(3)	–35.1	84.3
5-Methylfurfurylamine + phenol → 2-aminophenol+ 2,5-dimethylfuran	(4)	–34.4	79.9
5-Methylfurfurylamine + formaldehyde → furaldehyde + ethylamine	(5)	–11.5	78.6
5-Methylfurfurylamine + formaldehyde → methylamine + 5-methylfuraldehyde	(6)	–30.8	80.8
5-Methylfurfurylamine + benzene → 2,6-dimethylaniline + furan	(7)	–16.6	82.5
		$\langle \Delta_f H^o_m(g) \rangle$ (G3) = (–81.9 ± 0.9) kJ·mol^{-1}[a]	

[a]Mean and standard deviation.

Table S6. G3 computed enthalpies for furfurylamine and 5-methylfurfurylamine and for the auxiliary molecules used in the gas-phase reactions and standard molar enthalpies of formation, at T = 298.15 K, taken from the literature.

**Compound**	–G3<br>Enthalpy (T = 298.15 K) / a.u.[a]	$\Delta_f H_m^o$(g) / kJ·mol^{-1}
Furfurylamine	324.442123[b]	–43.5 ± 1.4[c]
5-Methylfurfurylamine	363.720635[b]	–81.2 ± 1.7[c]
Benzene	232.04673	82.9 ± 0.9 [1]
Aniline	287.368547	87.1 ± 1.1 [2]
2,6-Dimethylaniline	365.91845	18.6 ± 1.5 [3]
Benzaldehyde	345.31516	–36.7 ± 2.8 [2]
Toluene	271.321035	50.1 ± 1.1 [1]
Benzamide	400.661539	–100.9 ± 1.2 [4]
Methane	40.45381	–74.8 ± 0.34 [5]
Ethane	79.718911	–84.0 ± 0.4 [5]
Methylamine	95.75736	–23.4 ± 1.0 [2]
Ethylamine	135.028174	–47.5 ± 0.6 [2]
Formaldheyde	114.427242	–108.6 ± 0.5 [2]
Furan	229.855245	–34.8 ± 0.7 [2]
2-Methylfuran	269.133828	–76.4 ± 1.2 [6]
2,5-Dimethylfuran	308.412174	–123.5 ± 1.0 [7][d]
Furaldehyde	343.124092	–151.0 ± 4.6 [2]
5-Methyl-2-furaldehyde	382.403866	–196.8 ± 1.8 [6]

[a] All energies are in Hartrees. (1 Hartree = 2625.5 kJ·mol^{-1}); [b] Calculated considered the thermal populations in table S5; [c] This work; [d]L.M.P.F. Amaral, Calvet determination of enthalpy of vaporization, personal communication (2004) .

Table S7. Purification details of furfurylamine and 5-methylfurfurylamine.

Chemical name	CAS	Provenance	Initial mass fraction purity	Purification method	Final mass fraction purity	Analysis method
2-Furfurylamine	617-89-0	Sigma-Aldrich®	0.99	Distillation	0.99998	CO_2 recovery
5-Methyl-2-furfurylamine	14003-16-0	Sigma-Aldrich®	0.97	Distillation	0.99984	CO_2 recovery

Table S8. Standard molar heat capacities, in the gaseous phase, of furfurylamine and 5-methylfurfurylamine derived from statistical thermodynamics using the vibrational frequencies calculated at the basis B3LYP/6-31G(2df,p) level of theory (scaled by a factor of 0.965)[a] [8].

T / K	$C^{o}_{p,m}$ (g) / J·K^{-1}·mol^{-1}	
	Furfurylamine	5-Methylfurfurylamine
100	52.79	65.69
150	64.05	81.85
200	77.75	98.62
250	93.85	117.16
298.15	110.43	136.04
300	111.07	136.77
350	128.13	156.35
400	144.21	175.01
450	158.92	192.31
500	172.17	208.07
550	184.02	222.32
600	194.64	235.20

[a] The C_p of each conformer was obtained considering the Boltzmann relative population.

$C^{o}_{p,m}$ (g) =f(T), determined used to correct $\Delta^{g,T}_{l,298.15K}H^{o}_{m}$ to 298.15 K, using the equation:

$$\Delta^{g}_{l}H^{o}_{m}(298.15K)=\Delta^{g,T}_{l,298.15K}H^{o}_{m} - \int^{T}_{298.15\ K} C^{o}_{p,m}\ (g)dT \qquad (S1)$$

Furfurylamine

$$\frac{C^{o}_{p,m}}{J\cdot mol^{-1}\cdot K^{-1}} = -7.72952\times10^{-7}(T/K)^{3}+ 7.42328\times10^{-4}(T/K)^{2}+9.61589\times10^{-2}(T/K)+35.913 \qquad (S2)$$

5-Methylfurfurylamine

$$\frac{C^{o}_{p,m}}{J\cdot mol^{-1}\cdot K^{-1}} = -6.45289\times10^{-7}(T/K)^{3} + 5.82130\times10^{-4}(T/K)^{2}+2.09057\times10^{-1}(T/K)+39.237 \qquad (S3)$$

References

1. Roux, M.V.; Temprado, M.; Chickos, J.S.; Nagano, Y. Critically Evaluated Thermochemical Properties of Polycyclic Aromatic Hydrocarbons. *J. Phys. Chem. Ref. Data*, **2008**, *37*, 1855–1996.
2. Pedley, J.B.; Naylor, R.D.; Kirby, S.P. Thermochemical Data of Organic Compounds; Chapman and Hall: New York, NY, USA, 1986; pp. 1–792.
3. Verevkin, S.P. Thermochemical study of the ortho interactions in alkyl substituted anilines. *J. Chem. Thermodyn.*, **2000**, *32*, 247–259.
4. Gomez, L.A.T.; Sabbah, R. Thermodynamique de substances azotees. IX. Etude thermochimique de la benzamide. Comparaison des grandeurs energetiques liees a la structure de quelques amides et thioamides. *Thermochim. Acta*, **1982**, *58*, 311–315.
5. Manion, J.A. Evaluated Enthalpies of Formation of the Stable Closed Shell C1 and C2 Chlorinated Hydrocarbons. *J. Phys. Chem. Ref. Data*, **2002**, *31*, 123–172.
6. Ribeiro da Silva, M.A.V.; Amaral, L.M.P.F. Standard molar enthalpies of formation of some methylfuran derivatives. *J. Therm. Anal. Calorim*. **2010**, *100*, 375–380.
7. Verevkin, S.P.; Welle, F.M. Thermochemical Studies for Determination of the Standard Molar Enthalpies of Formation of Alkyl-Substituted Furans and Some Ethers. *Struct. Chem.*, **1998**, *9*, 215–221.
8. NIST Computational Chemistry Comparison and Benchmark Database. 2013. NIST Standard Reference Database Number 101, Release 16a; Johnson, R.D., III, Ed.; NIST Chemistry Webbook. SRD 69. Available online: http://cccbdb.nist.gov/vibscalejust.asp (accessed on 23 May 2023).
